# Metastatic Crohn’s Disease and Acute Myeloid Leukemia: A Diagnostic Paradox in a Severe Hyperinflammatory State

**DOI:** 10.7759/cureus.109424

**Published:** 2026-05-22

**Authors:** Francisco David Roman Delgado, María del Carmen Rivera García, Missael Isaac Ruiz Godinez, Ana Elena Escorcia Saucedo, Giovanni Elivt De La Rosa Patlan, Juan Carlos Anda Garay

**Affiliations:** 1 Internal Medicine, Hospital de Especialidades del Centro Médico Nacional Siglo XXI Instituto Mexicano del Seguro Social (IMSS), Ciudad de México, MEX

**Keywords:** acute myeloid leukemia, bicytopenia, crohn's disease, extraintestinal manifestations, hemophagocytic lymphohistiocytosis, infliximab, metastatic crohn's disease, myelodysplastic syndrome

## Abstract

Crohn's disease (CD) is a chronic immune-mediated inflammatory disorder in which extraintestinal manifestations occur in up to 40% of patients. Metastatic Crohn's disease (MCD), characterized by noncaseating cutaneous granulomas anatomically remote from the gastrointestinal tract, is among the rarest of these manifestations. Its coexistence with occult acute myeloid leukemia (AML) and secondary hemophagocytic lymphohistiocytosis (sHLH) in a treatment-naïve patient constitutes an exceptional clinical triad with profound diagnostic and therapeutic implications.

We report a 56-year-old woman without prior immunosuppressive exposure who presented with severe panenteric CD, MCD manifesting as necrotic lower-extremity nodules, and a submandibular abscess. Profound bicytopenia (hemoglobin: 5.3 g/dL, reference range: 13-18 g/dL; platelets: 12,000/μL, reference range: 150,000-450,000/μL) prompted a bone marrow evaluation, which revealed hypercellularity with 9% myeloid blasts (CD34+, CD117+). Although these findings required an urgent repeat marrow evaluation, cytogenetic/molecular testing, and hematology clearance due to the high risk of an indeterminate or pre-leukemic state, the presence of active, refractory gastrointestinal bleeding prompted the immediate initiation of infliximab induction. Given the absence of prior therapy and the presence of marked systemic inflammation, including concurrent pneumonia and active CD, these findings were initially attributed to reactive myelodysplastic mimicry. However, while systemic inflammation from pneumonia and active CD can mimic myelodysplasia, a 9% blast count with severe cytopenias should not be safely dismissed as merely reactive. Within days of the first infliximab infusion, the patient developed explosive leukocytosis (40,000/μL). Repeat flow cytometry demonstrated 37% immature myeloid blasts (CD34+, CD117++, HLA-DR+, CD123+), establishing a diagnosis of AML without maturation (FAB M1). Concurrently, overt hemophagocytosis with extreme hyperferritinemia confirmed concomitant sHLH (HScore: 227). This case highlights a critical and underrecognized risk: TNF-α may act as an endogenous immunological brake against clonal expansion. In our patient, TNF-alpha blockade may have contributed to the loss of immune control over an occult myeloid clone, dismantling this surveillance mechanism and precipitating a rapid transition to fulminant leukemia while triggering sHLH. Although causality cannot be proven from this single case and a direct triggering effect by infliximab remains speculative unless other potential triggers are thoroughly evaluated, the temporal association is striking. Severe systemic inflammation can create a reactive bone marrow phenotype indistinguishable from early myeloid neoplasia or myelodysplastic syndrome, posing a life-threatening diagnostic trap. Comprehensive hematologic evaluation-including repeat bone marrow assessment under controlled inflammatory conditions-should be considered mandatory before initiating biologic therapy in patients with CD and unexplained cytopenias or borderline blast counts.

## Introduction

Crohn's disease (CD) is a chronic, immune-mediated inflammatory disorder characterized by transmural gastrointestinal inflammation and a broad spectrum of extraintestinal manifestations (EIMs), affecting up to 40% of patients [1,2]. Among these manifestations, metastatic Crohn's disease (MCD), defined as noncaseating granulomatous inflammation of the skin in sites anatomically discontinuous from the gastrointestinal tract, is a rare and frequently underdiagnosed entity whose pathogenesis remains incompletely understood, with emerging evidence implicating the interleukin (IL)-23/T-helper (Th)17 axis in both intestinal and cutaneous granuloma formation [1,2]. The hematologic landscape of CD is similarly complex. While anemia is common, severe refractory bicytopenia warrants a broader differential diagnosis that includes drug-induced suppression, autoimmune cytopenias, and primary bone marrow disorders [3]. Importantly, chronic inflammatory states can drive reactive bone marrow changes, including transient dysplasia and hypercellularity, that closely mimic myelodysplastic syndromes (MDS), creating substantial diagnostic uncertainty [3]. Although IBD has been epidemiologically linked to an increased risk of MDS and acute myeloid leukemia (AML) [4,5], current evidence indicates that this association is largely mediated by cumulative immunosuppressive exposure rather than by the inflammatory disease per se [5], making clonal hematologic disease in treatment-naïve patients a particularly uncommon and diagnostically confounding event [3]. Superimposed on this complexity, secondary hemophagocytic lymphohistiocytosis (sHLH), a life-threatening hyperinflammatory syndrome driven by uncontrolled macrophage activation, can be triggered simultaneously by infections, hematologic malignancies, and autoimmune conditions [6]. Its occurrence in IBD, while rare, has been disproportionately reported during biologic induction, suggesting that immune dysregulation inherent to severe CD may create conditions permissive for hyperferritinemic collapse [3]. We present what we believe to be an exceptional case of a treatment-naïve patient with severe panenteric CD and MCD who, following infliximab induction for refractory disease, rapidly evolved to overt AML and sHLH conditions harbored silently beneath a florid inflammatory phenotype. This case illustrates the diagnostic pitfalls of borderline bone marrow findings in hyperinflammatory states and argues for mandatory hematologic clearance before biologic escalation in patients with CD and unexplained cytopenias.

## Case presentation

A 56-year-old woman with a history of acute myocardial infarction (revascularized in 2019), systemic arterial hypertension, and prediabetes presented with a one-month history of rhinorrhea, intermittent fever, night sweats, and weight loss. She subsequently developed painful oral ulcers, a right submandibular abscess, and ulcerated nodular skin lesions with necrotic centers involving the lower extremities and gluteal region (Figure 1). On admission, she was hemodynamically stable. Laboratory evaluation revealed a marked systemic inflammatory response (C-reactive protein 279 mg/L, ferritin 5908 ng/mL) and severe cytopenias (hemoglobin 5.3 g/dL; platelets ranging from 12,000/µL to 20,000/µL). Broad-spectrum antibiotics were initiated. Given suspicion for a granulomatous or vasculitic disease, an incisional skin biopsy was performed, demonstrating noncaseating granulomatous inflammation and multinucleated giant cells. Microbiological stains were negative.

**Figure 1:**
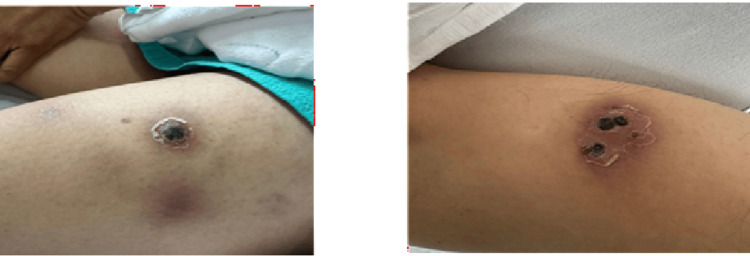
Skin lesions in lower extremities of the patient Multiple erythematous to violaceous plaques and nodules with central necrosis and bullous changes. As secondary features, peripheral desquamation and perilesional erythema are observed.

Endoscopic evaluation revealed erosive gastropathy and ulcerations involving the esophagus, ileum, and colon (Figure 2). The initial diagnostic approach focused on stabilizing suspected panenteric Crohn's disease and characterizing the cutaneous lesions, which were confirmed on biopsy as noncaseating granulomatous inflammation. Cross-sectional imaging showed nonspecific findings; however, contrast-enhanced CT of the abdomen and pelvis demonstrated segmental stenoses at múltiple levels of the gastrointestinal tract, supporting the diagnosis of extensive CD involvement (Figure 3). Behçet's disease was ruled out based on a negative pathergy test and absence of the HLA-B51 marker gene. Additionally, there was no evidence of cytomegalovirus infection.

**Figure 2:**
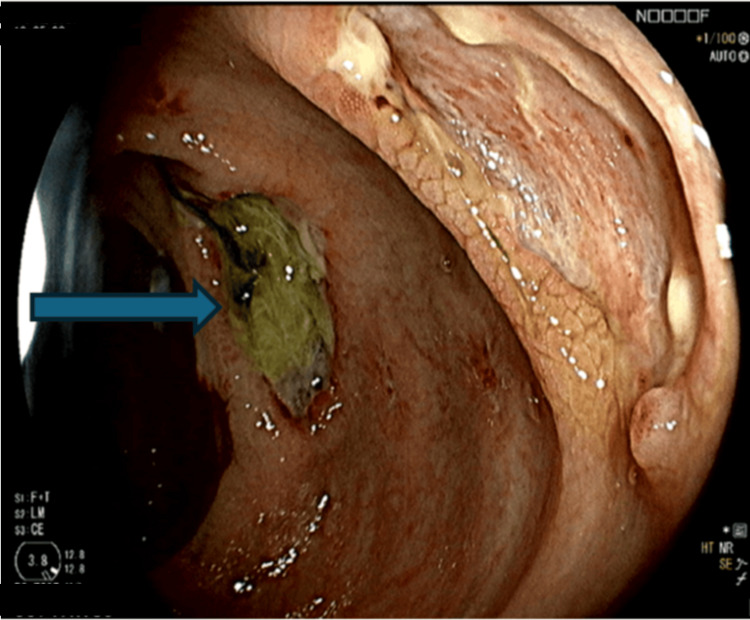
Upper and lower gastrointestinal endoscopic images of the patient Upper and lower gastrointestinal endoscopic findings suggestive of panenteric Crohn’s disease. Ileocolonoscopy displaying severe mucosal inflammation characterized by a large, deep, and irregular ulcer covered by a thick yellowish fibrinous exudate. The surrounding mucosa appears markedly edematous, hyperemic, and friable. These endoscopic findings are highly suggestive of an active, severe panenteric Crohn's disease, correlating with the patient's profound systemic inflammatory state and recurrent gastrointestinal bleeding.

**Figure 3:**
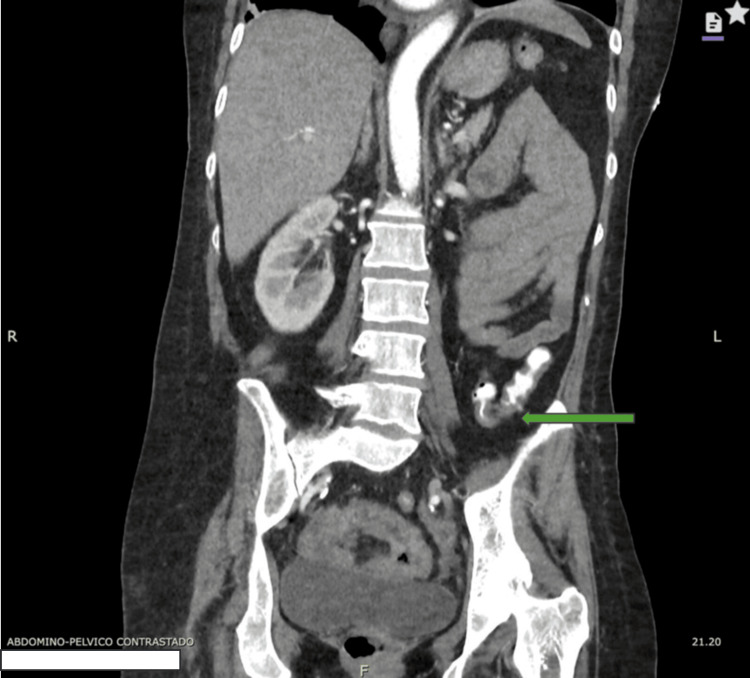
Contrast-enhanced abdominal-pelvic computed tomography (CT) image (coronal view) The image demonstrates severe intestinal stricture. The green arrow highlights a bowel segment in the left lower quadrant exhibiting stenosis.

Due to severe bicytopenia (Hb 5.3 g/dL, Platelets 12,000/µL), a bone marrow aspirate was performed, revealing hypercellularity with 9% myeloid blasts (CD45low, CD34+, CD117+). In the absence of prior thiopurine exposure and in the setting of a marked inflammatory state (pneumonia and active CD), these findings were initially interpreted as reactive changes (Table 1). However, persistent life-threatening gastrointestinal bleeding and refractoriness to corticosteroids prompted the multidisciplinary team to initiate infliximab after control of the infectious source. Shortly after induction, the patient developed marked leukocytosis.

**Table 1:** Evolution of key laboratory parameters ANA: Antinuclear antibodies. ANCA: Antineutrophil cytoplasmic antibodies. CRP: C-reactive protein. Hb: Hemoglobin. HBV: Hepatitis B virus. HCV: Hepatitis C virus. HIV: Human immunodeficiency virus. N/A: Not available. The table illustrates the persistent severe systemic inflammatory state (elevated CRP and escalating leukocytosis) inversely correlating with the refractory bicytopenia, supporting the hypothesis of inflammation-induced myelodysplastic mimicry over a primary bone marrow failure.

Date (2026)/Reference ranges	Hemoglobin (g/dL)/(13-18 g/dL)	Platelets (×10³/µL)/(150-450 ×10³/µL)	Leukocyte s (×10³/µL)/(4.6-10.2 ×10³/µL)	CRP (mg/L)/(<5 mg/L)	Ferritin (ng/mL)/(4.9-232 ng/ml)	Clinical context/Remarks and Specific workout
Jan 30	8.3	62	10.7	312	5908	Admission. Viral panel (HIV, HBV, HCV): Negative. ANA: Negative, p-ANCA & c-ANCA: Negative
Feb 11	8.3	24	13-8	259		GI workup: Fecal calprotectin: Positive. GI multiplex PCR panel: Negative.
Feb 16	5.3	12	11	279		Nadir of Hb and platelets
Mar 17	8	43	19	N/A		
Mar 24	7.7	62	16.83	N/A		Infliximab start
Mar 26	7.4	31	16.2	52.6		
Apr 01	6	25	40.49	170		
Apr 08	7.3	18	26	146	8371	Triglycerides: 190 mg/dL

An urgent repeat bone marrow evaluation with flow cytometry demonstrated 37% blast with low CD45 expression, positive for CD34, strongly positive for CD117, CD105, CD123, HLA-DR, CD13, CD33, and CD38, and negative for cytoplasmic myeloperoxidase and lymphoid markers. These findings established the diagnosis of AML without maturation (FAB M1). Concomitantly, bone marrow examination revealed overt hemophagocytosis (Figure 4). In the setting of extreme hyperferritinemia, this supported the diagnosis of secondary HLH with an HScore of 227.

**Figure 4:**
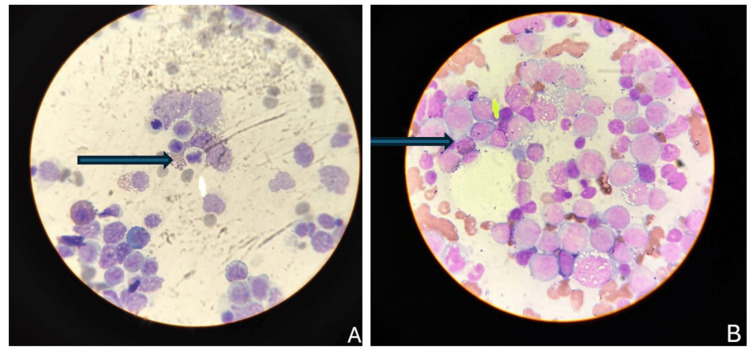
Bone marrow aspirate morphology (A) Micrograph (Wright-Giemsa stain, 100x magnification) showing a large, activated macrophage with ingested precursors and cellular debris, characteristic of hemophagocytosis in secondary hemophagocytic lymphohistiocytosis (sHLH). (B) Representative field (Wright-Giemsa stain, 100x magnification) showing hypercellular bone marrow with a monotonous infiltration of myeloid blasts exhibiting reticular chromatin, a high nucleus-to-cytoplasm ratio, and prominent nucleoli. No evidence of myeloid maturation is observed, highly suggestive of myeloid leukemia.

While initially considered reactive mimicry, the bone marrow karyotype, performed only after AML evolution, demonstrated a complex cytogenetic profile. This profile, characterized by monosomy 5, chromosome 7 rearrangement involving t(3;7)(q21;q36), trisomy 8, chromosome 12p abnormalities, trisomy 13, and a marker chromosome, confirmed the presence of an underlying high-risk clonal myeloid neoplasm that was not captured earlier in the diagnostic timeline.

Histopathological findings

Skin biopsy (January 9, 2026): Histopathological examination revealed a dense mixed inflammatory infiltrate, composed of acute and chronic inflammatory cells, nonspecific in nature. Multinucleated giant cells were identified, along with noncaseating granulomatous inflammation. No evidence of malignant cells was observed.

Endoscopic biopsy specimens (February 10, 2026): Stomach-mild chronic gastritis without evidence of atrophy or inflammatory activity; no Helicobacter pylori identified; Terminal ileum-ulcer bed; Ileocecal valve-ulcer bed with residual mucosa demonstrating chronic inflammation and regenerative epithelial changes; Ascending colon-acute and chronic colitis; Transverse colon-chronic colitis with moderate inflammatory activity; Descending colon-chronic colitis with mild inflammatory activity; Rectum-focal acute and chronic proctitis.

Bone marrow biopsy (March 4, 2026): Bone marrow with evaluable intertrabecular spaces, demonstrating approximately 98% cellularity. The erythroid lineage shows normoblastic maturation. The myeloid lineage exhibits limited maturation to band and segmented forms. There is an increased proportion of cells with blast morphology. Diagnosis: Increased proportion of cells with myeloid blast morphology, consistent with myeloid leukemia. No immunohistochemistry was available at the institution.

Flow cytometry immunophenotype (March 3, 2026): Bone marrow flow cytometry demonstrated dysplastic changes within the myeloid and monocytic compartments, with aberrant maturation patterns and CD123 expression. An abnormal blast population (~9%) was identified, characterized by CD34+, CD117+, HLA-DR+, CD13+, and CD33+, with low CD45 expression. Blasts lacked lymphoid markers (CD19, CD3, TdT) and showed no clear monocytic differentiation (CD14 negative). Interpretation: Findings indicate aberrant myeloid maturation with increased myeloid blasts.

Bone marrow findings and hematology evaluation: The bone marrow aspirate (March 4, 2026) demonstrated approximately 80% cellularity, although interpretation was limited by a reactive background. Mild dysplastic changes (<10%) were identified in the myeloid lineage, including karyorrhexis, binucleated erythroid precursors, and nuclear-cytoplasmic asynchrony. Myeloid hyperplasia with hypergranular promyelocytes and myelocytes was observed. Approximately 5% of cells were immature, with no definitive evidence of clonal blasts. Flow cytometry revealed myeloid and monocytic maturation asynchrony, with an immature population comprising 9% of total cells. Overall, these findings suggest multifactorial cytopenias in the context of systemic inflammation, with a possible underlying myelodysplastic process.

Bone marrow evaluation (April 7, 2026): Bone marrow aspirate showed mild hypercellularity for age, with 0-1 dysplastic megakaryocytes per high-power field and marked erythroid dysplasia. Normal-appearing plasma cells, eosinophils, and numerous macrophages were observed, several exhibiting hemophagocytosis. Conclusion: Findings are compatible with reactive hemophagocytic lymphohistiocytosis, with no evidence of acute malignant neoplasia identified by this method.

Flow cytometry immunophenotype (April 7, 2026): Bone marrow flow cytometry revealed 37% blasts with an immature myeloid phenotype characterized by CD34+, CD117++, HLA-DR+, CD13+, CD33+, CD38+, CD105+, and CD123+, with low CD45 expression. Blasts were negative for lymphoid markers (CD19, CD3, cyCD3, CD79a, TdT) and monocytic markers (CD14, CD64), as well as myeloperoxidase (cyMPO). Interpretation: Findings support the diagnosis of AML without maturation (FAB M1).

Bone marrow evaluation (April 9, 2026): A reassessment of the bone marrow was performed in conjunction with the hematopathology service. The specimen was mildly hypercellular for the patient’s age, showing 0-1 megakaryocytes per high-power field with dysplastic features. Marked erythroid dysplasia was observed. Additionally, myeloid blasts with morphology consistent with FAB M1 subtype were identified, comprising up to 80% of nucleated cells. Conclusion: Findings are consistent with acute myeloid leukemia.

Bone marrow karyotype (April 14, 2026): 48,XX,-3,-5,der(7)t(3;7)(q21;q36),+8,+8,add(12)(p13),+13,+mar/45,XX,-3.-5,t(3;7)(q21;q36),+8,del(12)(p12).

## Discussion

The clinical trajectory described in this report encapsulates a diagnostic and therapeutic paradox of exceptional rarity: a treatment-naïve patient in whom the biological therapy selected to control a life-threatening inflammatory condition simultaneously dismantled the endogenous immune surveillance restraining an occult leukemic clone. The simultaneous coexistence of metastatic Crohn's disease, acute myeloid leukemia, and secondary hemophagocytic lymphohistiocytosis-each individually rare, each mutually amplifying-constitutes, to our knowledge, a previously unreported clinical triad with profound implications for the management of severe inflammatory bowel disease complicated by unexplained cytopenias.

Metastatic Crohn's disease, defined by noncaseating granulomatous inflammation at sites in the skin anatomically remote from the gastrointestinal tract, is among the rarest and most diagnostically elusive extraintestinal manifestations of CD [1,2]. Its pathogenesis is incompletely characterized, though current evidence increasingly implicates the IL-23/Th17 axis as the central immunological driver of both intestinal and cutaneous granuloma formation [1]. The broad and overlapping differential diagnosis, encompassing vasculitis, sarcoidosis, pyoderma gangrenosum, and cutaneous infections, frequently contributes to diagnostic delays, as was initially the case in our patient [1,2]. Confirmation ultimately required histopathological demonstration of noncaseating granulomatous inflammation with sterile microbiological cultures.

The central diagnostic dilemma of this case was the interpretation of 9% myeloid blasts in the context of severe systemic inflammation. Current evidence establishes that chronic inflammatory states, recurrent infections, and pathological cytokine signaling can generate reactive bone marrow changes, including transient dysplasia, myeloid hyperplasia, and increased immature precursors, that are morphologically and immunophenotypically indistinguishable from early myelodysplastic neoplasia [3]. Crucially, WHO diagnostic criteria for MDS mandate the exclusion of reactive etiologies prior to establishing a clonal diagnosis, providing the conceptual and practical framework within which our initial interpretation of reactive myelodysplastic mimicry was both defensible and methodologically appropriate [3,4]. The absence of prior immunosuppressive exposure in our patient further reinforced this interpretation: population-based analyses have demonstrated that the excess risk of myeloid neoplasms in IBD patients attenuates to statistical non-significance after adjusting for cumulative exposure to thiopurines and anti-TNF agents [5,6], suggesting that the inflammatory disease per se is a considerably weaker leukemogenic driver than its pharmacological management.

The explosive leukemic evolution following infliximab induction highlights a critical, underrecognized immunobiological risk. Tumor necrosis factor (TNF)-α occupies a dual position in the tumor microenvironment: while it mediates inflammatory tissue injury in diseases such as CD, it simultaneously coordinates cytotoxic immune surveillance of malignant and pre-malignant cells through direct apoptotic signaling and activation of natural killer cell and T-lymphocyte responses [7]. In patients harboring a pre-leukemic clone, endogenous TNF-α may therefore function as an immunological restraint on clonal expansion-a brake that, when pharmacologically neutralized, permits unchecked leukemic transition. The temporal correlation between infliximab induction and the rapid progression from 9% to 37% blasts in our patient is consistent with this mechanism [7] and is corroborated by published reports of AML development, including acute promyelocytic leukemia, shortly after initiating anti-TNF therapy in patients with CD [8,9]. At the population level, meta-analytic data from randomized controlled trials confirm that anti-TNF therapy is associated with a more than threefold increase in malignancy risk (OR 3.3; 95% CI, 1.2-9.1), with the highest excess risk observed at induction doses [10], precisely the clinical scenario encountered in our patient.

The severe infectious burden sustained by our patient, concurrent pneumonia and a submandibular abscess superimposed on an active CD flare, likely functioned as an additional pathological amplifier, driving pro-inflammatory cytokine release that simultaneously promoted clonal expansion and activated macrophage hyperactivation pathways [3]. This infectious stimulus, combined with anti-TNF-mediated immunosuppression, generated the hyperinflammatory milieu necessary for the emergence of secondary HLH. A systematic review by Coburn et al. documented that HLH in IBD occurs predominantly in CD (83% of cases) and that 34.5% of episodes arise during biologic induction [11], a pattern strikingly concordant with our case. As established by Henter et al., the classical triggers of sHLH are infections, hematologic malignancies, and autoimmune conditions [12]; our patient harbored all three concurrently, creating a uniquely permissive environment for macrophage hyperactivation. The HScore of 227, corresponding to a diagnostic probability exceeding 93%, provided objective confirmation of sHLH [13] in a clinical context where hyperferritinemia alone would have been insufficient, given its well-recognized lack of specificity in the setting of active inflammatory disease [11,12].

The therapeutic decision-making process in this case merits careful analysis, as it reflects a broader clinical dilemma likely to recur as biologic therapy becomes increasingly prevalent in severe IBD. Ustekinumab, an IL-12/23 inhibitor, represented a theoretically superior alternative: its safety profile with respect to serious infections is favorable compared to anti-TNF agents [14], it targets the IL-23/Th17 pathway implicated in both intestinal and cutaneous granuloma formation in MCD [1,15], and it has demonstrated efficacy in IBD associated with myelodysplastic processes, offering potential hematological benefit without the leukemogenic risk of TNF-α blockade [15]. However, the clinical urgency imposed by recurrent life-threatening gastrointestinal bleeding, combined with the more established evidence base for infliximab in acute severe luminal CD, led the multidisciplinary team to prioritize rapid disease control [14]. The decision was further informed by evidence that TNF-α mediates premature apoptosis of hematopoietic precursors in MDS, and that infliximab has been investigated as a strategy to improve hematopoiesis in low-risk MDS [16], a rationale that, while biologically coherent, did not account for the possibility that the marrow blasts represented not a reactive MDS phenotype but an occult leukemic clone in which TNF-α suppression would prove catastrophic.

This case generates several lessons with direct clinical applicability. First, severe systemic inflammation can produce a reactive bone marrow phenotype, including increased blasts and dysplastic changes, that is morphologically and immunophenotypically indistinguishable from early myeloid neoplasia, making repeat evaluation under controlled inflammatory conditions essential before any definitive hematological diagnosis is established or excluded [3]. Second, in patients with CD and unexplained cytopenias or borderline blast counts, the initiation of potent immunosuppression, particularly anti-TNF induction, must be preceded by a comprehensive multidisciplinary hematological assessment, including repeat bone marrow evaluation whenever clinically feasible [3,14]. Third, this case provides clinical evidence supporting the hypothesis that endogenous TNF-α serves as an immunological restraint on pre-leukemic clonal expansion, and that its pharmacological neutralization may precipitate fulminant leukemic transformation in susceptible patients [7,10]. Fourth, the simultaneous presence of infection, hematologic malignancy, and autoimmune disease-all established triggers of sHLH-should prompt early application of validated diagnostic criteria such as the HScore [13], even in the absence of a confirmed trigger, as diagnostic delay in sHLH is independently associated with mortality [11,12]. Future research should prioritize the identification of molecular biomarkers capable of distinguishing reactive myelodysplastic mimicry from true clonal evolution in hyperinflammatory states, and the prospective evaluation of the hematological safety of anti-TNF agents in patients with IBD and concurrent cytopenias. Until such data are available, a structured multidisciplinary approach integrating gastroenterology, hematology, and hematopathology represents the minimum standard of care in this exceptional but increasingly relevant clinical scenario [3,7,14].

## Conclusions

The coexistence of severe Crohn's disease with unexplained cytopenias and borderline bone marrow blasts represents an exceptionally high-risk clinical scenario. This case serves as a stark warning: while anti-TNF agents are essential for inducing remission in severe inflammatory bowel disease, they can inadvertently disrupt vital immune surveillance. In patients with intense inflammation, this immunosuppression can act as a molecular catalyst, unmasking a hidden malignant clone and triggering its fulminant progression to overt acute myeloid leukemia and secondary hemophagocytic lymphohistiocytosis. Therefore, in patients with Crohn's disease who present with unexplained cytopenias or extreme hyperferritinemia, clinicians must implement a highly structured, multidisciplinary diagnostic approach. A comprehensive bone marrow evaluation to rule out primary malignancies is essential before initiating potent immunosuppression. Future research should focus on identifying reliable molecular biomarkers capable of distinguishing reactive myelodysplastic mimicry from true clonal evolution in hyperinflammatory states.

Currently, there are insufficient studies to confirm the hypothesis of an "immunological brake" provided by TNF-α, which, when blocked, triggers blast proliferation. Therefore, it is crucial to investigate the hematological safety of anti-TNF-α biologics in patients susceptible to hematological malignancies and the efficacy of alternative targeted therapies, such as IL-12/23 inhibitors, in this vulnerable population. Diagnostic improvement remains a critical unmet need. Importantly, subsequent cytogenetic analysis revealed a complex karyotype including monosomy 5, chromosome 7 abnormalities involving 3q21 rearrangement, trisomy 8, and 12p alterations, strongly supporting the presence of an underlying clonal myeloid neoplasm rather than purely reactive inflammatory dysplasia. These findings suggest that the patient likely harbored an occult high-risk myeloid process during the initial inflammatory presentation, with severe systemic inflammation and active Crohn´s disease obscuring early diagnostic interpretation.
